# Comparison of postoperative pain intensity following the use of three different instrumentation techniques: A randomized clinical trial

**DOI:** 10.15171/joddd.2019.021

**Published:** 2019-08-14

**Authors:** Mehmet Adiguzel, Pelin Tufenkci, ismail Ilker Pamukcu

**Affiliations:** ^1^Department of Endodontics, Faculty of Dentistry, Mustafa Kemal University, Hatay, Turkey

**Keywords:** Endodontics, instrumentation, postoperative pain, root canal therapy

## Abstract

***Background.*** The aim of this study was to compare the postoperative pain intensity following the root canal preparation carried out with XP-endo Shaper (XPS; FKG Dentaire SA, La Chauxde-Fonds, Switzerland), iRace (iRC; FKG Dentaire SA) and Reciproc Blue (REC Blue; VDW, Munich, Germany) files.

***Methods.*** Mandibular molar teeth with asymtomatic necrotic pulps in 69 patients were randomly divided into three groups (n=23). The root canals were prepared using XPS, iRC or REC Blue instruments and obturated using the lateral condensation technique. The patients were asked to record their pain intensity at 24-, 48- and 72-hour and 1-week postoperative intervals on VAS. For intolerable pain after the procedure, ibuprofen (400 mg) was prescribed. Data were analyzed using chi-squared, Friedman, Kruskal–Wallis, and Mann–Whitney U tests.

***Results.*** The postoperative pain gradually decreased during the study period in all the groups (P<0.05). No statistically significant difference was found between iRC system and the two other systems at 12-, 24- 48-hour and 1-week intervals (P>0.05). When compared to XPS system, a higher level of postoperative pain was observed with REC Blue system at 24- and 48-hour intervals (P<0.05).

***Conclusion.*** The XPS group exhibited less postoperative pain than the REC Blue group at 24- and 48-hour intervals. iRC, XPS and REC Blue systems were found to be similar in terms of postoperative pain severity.

## Introduction

One of the most frequently observed complications of root canal treatment is postoperative (post-op) pain. In the literature, there are studies reporting the prevalence of post-op pain after root canal treatment to vary between 3% and 58%.^1-3^ The occurrence of postoperative pain is related with insufficient root canal preparation, complications during the irrigation procedure, and the presence of periapical pathology.^4^ One of the most important reasons for postoperative endodontic pain is the extrusion of infected dentin, microorganisms and irrigation solutions from the root apex during the chemomechanical preparation. It damages the periradicular tissues and might cause an acute inflammatory response. Depending on the level of damage in periapical tissues, the postoperative pain might be at high or lower levels.^3^

The pain levels of the patients after the use of different preparation methods in root canal treatment have been examined in various studies.^5,6^ There is no consensus on which rotary and reciprocal system results in more extrusion and which one causes less.^7,8^ Despite the technological advancements, it was reported that different preparation methods and different file systems produced different levels of dentin extrusion.^3^ The factors such as file designs, tip diameter, taper and preparation method might affect the amount of apical extrusion.^4,6^

Thanks to advances in production methods and metallurgy, as well as innovative concepts, it became possible to manufacture systems offering easier and faster instrumentation.^6^ XP-Endo Shaper (XPS; FKG Dentaire SA, La Chauxde-Fonds, Switzerland), continuous rotary single file system combining the MaxWire and Booster Tip technologies, was introduced recently. XPS files are in the martensitic phase at room temperature because of their aluminum content. Moreover, when placed into the canal at room temperature, they enter the austenitic phase (memorized shape). With an initial taper of 1%, the XPS file turns from a straight shape to a serpentine shape. This serpentine shape pushes out the envelope of movement and the files achieve a taper of 4%.^9^ iRace (iRC; FKG Dentaire SA) system incorporates only 3 iRC NiTi rotary files in order to treat most of the cases. The files, for which an electrochemical surface polishing method is used, have a triangular cross-section and special anti-screw design. The file design allows prevention of the inward screwing effect and better control on the file progression.^9,10^ Reciproc Blue single file system (REC Blue; VDW, Munich, Germany), which operates based on the reciprocal motion and is an updated version of Reciproc (VDW, Munich, Germany) canal file, is now available on the market. REC Blue R25, R40 and R50 files are used for narrow, medium and wide canals, respectively. The REC Blue R25 (25/.08) files can be used in most of the cases. The geometry, size and design of REC Blue files are the same as those of Reciproc file system. REC Blue files are manufactured by using an innovative heat treatment method altering the molecular structure of the file. Specific S-shaped cross-section, variable taper, cutting angles and non-cutting tip of file increase the performance and efficiency.^11^

There are various studies reporting that the root canal treatment of a tooth with asymptomatic necrotic pulp can be completed in a single session or multiple visits.^1,12,13^ In some clinical studies, it was determined that patients preferred and better tolerated root canal treatment completed in a single visit.^13,14^ The single-visit root canal treatment was safer in both vital and non-vital teeth in terms of endodontic flare-ups.^13^ In the literature, there is no study available to have compared the postoperative pain severity after using three different file systems. The aim of the present study was to compare postoperative pain levels after using XPS (continuous rotary single file system), iRC (continuous rotary multi-file system) and REC Blue (reciprocating single file system) in single-visit root canal treatment. The null hypothesis of the present study was that the instrumentation system used does not affect the severity of postoperative pain.

## Methods

The approval of the Ethics Committee of Mustafa Kemal University was obtained for the present randomized clinical trial (IRB No. 2017/190). All the subjects included in the present study signed informed consent forms about the treatment, risks and advantages. In addition, the subjects participated in the study on a voluntary basis. The sample size was determined by taking the results of a pilot study as basis. Based on the results of the pilot study, the final sample size was set at 69 (23 subjects in each group) for the α-value of 0.05 and study power of 80%.

### Participants

**Figure 1:**
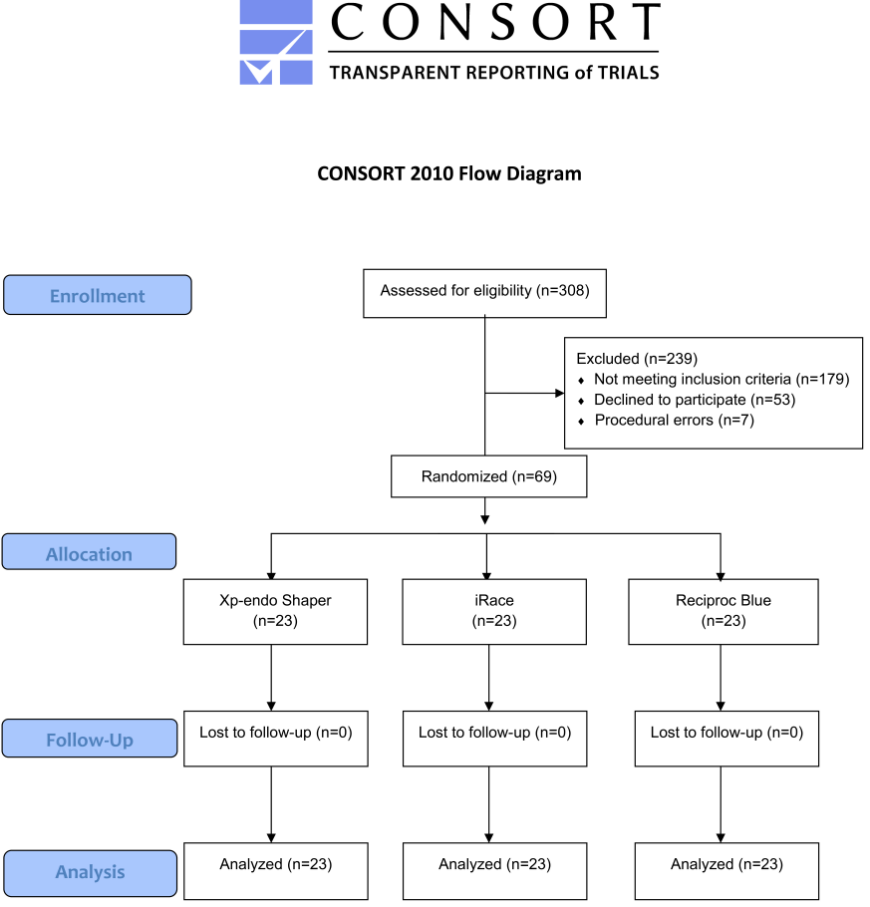
Flowchart.

The participants were selected from the patients referring to the Endodontics Department of Mustafa Kemal University from September 2017 to February 2018. In the present study, 69 patients aged 21‒65 years, with no periapical pathology but first or second mandibular tooth with asymptomatic necrosis, were evaluated. After intraoral examinations, the demographic data of the patients and the locations of teeth were recorded. The pulp vitality and periradicular status of each tooth were determined using thermal and electric pulp tests, followed by palpation, percussion and periodontal charting. The clinical diagnosis of asymptomatic pulp necrosis was made based on the negative response to Green Endo-ice cold test and the electric pulp test. The diagnosis was supported because no bleeding was observed in the root canals after opening an access cavity. The participants did not take any medication suppressing or changing pain perception such as antibiotics, narcotics, antidepressants and analgesics during the week before the procedure. Moreover, participants with periapical lesions, abscesses or cellulitis at the relevant tooth, those with a medical treatment history or those having undergone root canal treatment for the relevant tooth were excluded from the study. The study flowchart for inclusion in the present randomized controlled trial is shown in Figure 1.

### Randomization

In this process, a dark-colored bag was used; 23 blue, 23 yellow and 23 green papers were prepared for 69 participants. The colored papers were put in the bag and the bag was closed. Before the procedure, a dental assistant in the clinic was asked to randomly choose a paper from the bag. The treatments were completed by using XPS file for green color, iRC file for yellow paper and REC Blue file for blue color. The patients were assigned to 3 groups according to the instrumentation system used. All the participants involved in the study were informed about the used systems prior to the treatment.

A total of 7 patients were excluded from the study due to procedural errors and then another 7 patients were included in the study.

### Treatment Protocol

As in the similar studies carried out before, local anesthetic solution containing 1:100,000 epinephrine (Ultracain DS Forte; Sanofi-Aventis) and 4% articaine was used for the inferior alveolar nerve block.^6,15^ After anesthesia, a standard access cavity was prepared in the mandibular teeth and the root canal orifices were found using #10 manual K-ﬁles (VDW). The teeth were isolated by using a rubber dam. The working length was set to 1 mm shorter than the apical foramen by using #10 K-file and an apex locator (J. Morita Co., Tustin, CA) and then confirmed with periapical radiography. The working length was repeatedly checked throughout the treatment procedure. One of the three file systems was used for shaping and cleaning the root canals in a single visit. Each file was used only in a single tooth.

### XPS group

After determining the working length, the canal was shaped using #15 K-file (VDW). XPS (30/.01) files were used in continuous rotational movement with VDW Silver Reciproc endodontic motor at the speed and torque levels recommended by the manufacturer (800 rpm and 1 Ncm). 10 mL of 2.5% NaOCl was used in the root canals in total and the root canals were also irrigated with 17% EDTA before obturation.

### iRC Group

iRC files were operated in the rotational movement using the same endodontic motor in accordance with the recommendations of manufacturer (600 rpm at 1.5-Ncm torque). iRC systems were operated using a #15 file with 0.06 taper, #25 file with 0.04 taper and #30 file with 0.04 taper, respectively. After every file replacement, the root canals were irrigated using 2.0 mL of 2.5% NaOCl solution. 10 mL of 2.5% NaOCl was used in root canals in total and then the root canals were irrigated with 17% EDTA before obturation.

### REC Blue Group

The root canals were shaped at the working length by using #15 K-files (VDW). Then, REC Blue R25 (25/.08) files were used with the same endodontic motor in “Reciproc all” mode. The instrument was moved using a slow in-and-out pecking motion (not more than 3‒4 mm) with light apical pressure. 10 mL of 2.5% NaOCl was used in root canals in total and then the root canals were irrigated with 17% EDTA before obturation.

After the preparation procedures, the root canals were dried using paper points and then obturated by using cold lateral compaction method in the same visit. After radiologically confirming the obturation, the teeth were restored using a resin-modified glass-ionomer and a nano-hybrid composite resin.

### Pain Assessment

Visual analog scale (VAS) was used for pain assessment. VAS consists of a line marked from 1 to 10 and the pain level is categorized as no pain (0), mild pain (1–3), moderate pain (4–6) and severe pain (7–10). The scale was given to the patient and the patients were instructed verbally and visually about how to fill the form. Then, the patients were asked to record their pain at 24-, 48- and 72-hour and 1-week postoperative intervals. For intolerable pain after the procedure, ibuprofen (400 mg) was prescribed. At 24-, 48- and 72-hour and 1-week postoperative intervals, a researcher with no information about the present study recalled the patients and the pain scores were recorded. Moreover, the use of analgesics by the patients was recorded, too.

### Statistical Analysis

Data were analyzed with SPSS 21.0 (SPSS Inc., Chicago, IL). The normal distribution of data was examined with Shapiro-Wilk test. Friedman, Kruskal‒Wallis, and Mann‒Whitney U tests were used to compare the effects of different instrumentation systems on postoperative pain. Chi-squared test was used to compare the demographic data and use of analgesics.

## Results

Sixty-nine patients were included in the present study. No patient died during the follow-up period. The demographic data (age, sex and type of teeth) of patients are presented in Table 1, postoperative pain levels in Table 2 and use of analgesics in Table 3. No statistically significant differences were found between the groups in terms of demographic variables and analgesic usage (P>0.05). Postoperative pain gradually decreased during the study period in all the groups (P<0.05). For all the periods, the minimum level of postoperative pain was observed in the XPS group and the maximum level in the REC Blue group. No statistically significant differences were found between the iRC system and the two other systems at 12-, 24- and 48-hour and 1-week postoperative intervals (P>0.05). Statistically significant differences were found between the systems in terms of pain level at 24- and 48-hour intervals. When compared to the XPS system, a higher level of postoperative pain was observed with the REC Blue system at 24- and 48-hour intervals (P<0.05).

**Table 1:** Demographic data of each group

**Demographic Data**	**Xp-endo Shaper**	**iRace**	**Reciproc Blue**	P-value*
**Age**	38.16±10.42	36.44±12.94	39.74±13.78	P>0.05
**Male**	12	11	10	
**Female**	11	12	13	
**Lower 1. molar**	10	13	11	
**Lower 2. molar**	13	10	12	

**Table 2:** VAS scores of postoperative pain for each group

**Used system**	**No.**	**Mean**	**Median**	**SD**	**Minimum**	**Maximum**	P-value*
**Pain after 24 h**							0.010*
**Xp-endo Shaper**	23	0.95	0.00	1.42	0.00	4.00	
**iRace**	23	1.69	1.00	1.69	0.00	5.00	
**Reciproc Blue**	23	2.60	3.00	1.92	0.00	5.00	
**Pain after 48 h**							0.029*
**Xp-endo Shaper**	23	0.60	0.00	1.11	0.00	4.00	
**iRace**	23	1.17	1.00	1.26	0.00	4.00	
**Reciproc Blue**	23	1.43	1.00	1.19	0.00	3.00	
**Pain after 72 h**							0.134
**Xp-endo Shaper**	23	0.21	0.00	0.59	0.00	2.00	
**iRace**	23	0.56	0.00	1.12	0.00	4.00	
**Reciproc Blue**	23	0.73	0.00	1.05	0.00	3.00	
**Pain after 1 week**							0.283
**Xp-endo Shaper**	23	0.08	0.00	0.28	0.00	1.00	
**iRace**	23	0.26	0.00	0.54	0.00	2.00	
**Reciproc Blue**	23	0.30	0.00	0.55	0.00	2.00	

*Kruskal-Wallis test, P<0.05.

**Table 3:** Frequency of analgesic intake according to group

**Analgesic intake**	**Xp-endo Shaper**	**iRace**	**Reciproc Blue**	P-value*
	**Frequency (%)**	**Frequency (%)**	**Frequency (%)**	
** None**	13 (56.5%)	10 (43.5%)	9 (39.1%)	P>0.05
** 1 tablet**	7 (30.4%)	7 (30.4%)	8 (34.8%)	
** 2 tablets**	3 (13.0%)	4 (17.4%)	3 (13.0%)	
** 3 tablets**	0 (0.0%)	2 (8.7%)	3 (13.0%)	
** Total**	23 (100%)	23 (100%)	23 (100%)	

## Discussion

Postoperative pain might occur because of factors such as age, sex, pulpal and periradicular factors, preoperative pain and the implemented method.^4^ Among these factors, choosing a suitable preparation method and shaping the root canal are under the control of the physician. For this reason, severe postoperative pain might be prevented by shaping the root canal successfully, extruding the canal contents during the preparation procedure, and minimizing extrusion of this content over the periradicular tissues.

In the present study, several criteria were implemented in eliminating the effects of different preoperative factors. Similar preoperative pain levels were achieved by including only asymptomatic teeth in the study. Since the preoperative periapical condition has a significant effect on postoperative pain, only teeth with necrotic pulps without any periapical pathology were included in the present study. The present study was carried out on the mandibular molar teeth. It has been reported that these teeth have the highest incidence of postoperative pain.^16^ Other factors that might influence the amount of extruded debris (operator, irrigation, obturation) were standardized in all the groups. Ibuprofen, which is the first analgesic option offered to the patients in many studies the relief of pain after the root canal treatment, was prescribed.

The scale should be easily understood by the patients and clearly interpreted by the researchers in order to determine the pain level of patients objectively. For this purpose, the VAS scale which is widely preferred in the literature was employed in the present study.^1,17^

The single-visit endodontic treatments have become popular in recent years.^18^ Su et al^14^ reported that the incidence of pain after the single-visit endodontic treatment was less than that observed after the multiple-visit endodontic treatment. The apical extrusion of intracanal medications used in multiple-visit treatments, failure or leakage of temporary filling materials or the other factors that might affect pain might cause postoperative pain between visits. In the present study, the root canal treatments were completed in a single visit in order to minimize the effects of these factors.

The aim of this prospective, randomized controlled clinical trial was to compare the effects of three different root canal preparation systems on postoperative pain. For all the periods, the minimum level of postoperative pain was observed in the XPS group and the maximum level in the REC Blue group. For 24h and 48h periods, significantly higher levels of postoperative pain were observed in the REC Blue group when compared to the XPS group. For this reason, the null hypothesis of the present study was rejected.

The severity of postoperative pain was observed to decrease gradually at all the time intervals, in which the pain measurement was performed after the root canal treatment. In a systematic review carried out in 2011, Pak and White^19^ reported that the highest level of postoperative pain was observed in the early phase after root canal treatment. The postoperative pain incidence was reported to be 40% in the first 24 hours, to decrease significantly in the first 48 hours, and to be 11% or less on the 7th day. Similar results were also achieved in the present study.

It is very important to take the movement kinematics of file systems into consideration. Some studies showed that the postoperative pain level with the use of rotary file systems was significantly lower than that with the use of reciprocal single-file systems. The difference between the groups was attributed to extrusion of debris, occurring depending on the instrumentation method.^7,20,21^ Previous studies showed that one of the most important reasons for postoperative pain is the extrusion of debris in the canal from the tip of the root during chemomechanical preparation, and that this would cause peripheral sensitivity characterized by hyperalgesia and spontaneous pain.^6,22^ It was determined in the in vitro studies that, compared to the rotary file systems, the reciprocal file systems might cause higher amount of debris extrusion as a result of the reverse motion of instruments.^3,7^ During the rotary motion, the mechanical movement wave courses throughout the length of the working part of the instrument, minimizing the contact between the file and dentin. In this case, when compared to the reciprocal instruments, rotary files yield cleaner canals by ensuring lower amounts of debris accumulation.^23^ This was confirmed in the present study because the maximum postoperative pain was observed in the REC Blue reciprocal file group, whereas the minimum postoperative pain was found in the XPS rotary file group.

Another point to consider is to compare the number of files required for preparing the root canals. When compared to REC Blue or XPS single file systems, more files are used in root canal preparation performed using multiple-file iRC rotary file system. Longer duration of contact with the root canal walls might result in the formation of higher amounts of debris and higher level of manipulation in the apical region.^24^ Reaching to the working length by using higher number of instruments might cause more extrusion and postoperative pain. It can be thought that the higher level of postoperative pain observed with the iRC rotary file system when compared to XPS file system might be because of higher number of files, longer duration of preparation and possible increase in the amount of apical extrusion.

In the present study, the files were tested in the XPS (30/.01), iRC (30/.04) and REC Blue groups (25/.08). Lower level of postoperative pain was achieved in the XPS group with smaller size and taper, whereas a higher level of postoperative pain was achieved in the REC Blue group with larger taper. The differences in sizes might affect the results of the study by causing different amounts of extruded debris. Uslu et al^9^ compared the amounts of debris extruded from the apex during the root canal instrumentation by using XPS, HyFlex EDM and REC Blue files. According to the results of the present study, the REC Blue files produced significantly higher amounts of extruded debris compared to the XPS files. This conclusion was supported because the level of postoperative pain in the REC Blue group was higher than that in the XPS group.

Previous studies have shown that reciprocal files result in higher levels of debris extrusion compared to rotary files. Burklein et al^20^ asserted that Reciproc file system caused more debris extrusion compared to OneShape, F360 and Mtwo file systems. Similarly, Lu et al^25^ reported that use of Reciproc instruments in retreatment procedures led to higher amount of debris extrusion compared to Mtwo R instrument. On the other hand, Üstün et al^8^ compared the ProTaper Next, Twisted File and WaveOne systems in terms of the amount of debris extrusion and reported that WaveOne system resulted in less debris than the two other systems did. Shokraneh et al^6^ compared postoperative pain levels after the use of three different instrumentation methods in mandibular molar teeth with necrotic pulps. They reported that the root canal treatments performed using WaveOne system created significantly lower levels of postoperative pain compared to the root canal treatments performed using ProTaper Universal rotary system and hand files, and they attributed this to the amount of infected debris extruded from the apex. Neelakantan et al^22^ reported that Reciproc file system resulted in lower levels of postoperative pain when compared to OneShape file system. However, Comparin et al^18^ reported that Mtwo and Reciproc file systems were found to be equivalent in terms of postoperative pain levels. The possible explanation for the differences between the opinions on this topic is that the amount of infected debris extruded depends on the motion, number, design and taper of files.^26^

It should be noted that the size, cross-section, number of files and kinematics of the instrumentation systems used are different from each other, and these differences might have affected the results of this study.

## Conclusions

The XPS group exhibited less postoperative pain than the REC Blue group at 24- and 48-hour intervals. iRC system and XPS and REC Blue systems were found to be similar in terms of postoperative pain severity.

## Author Contributions

MA was responsible for conceptualization, data collection, formal analysis, funding acquisition, investigation, methodology, resources, software, supervision, validation, visualization, and writing the original draft and reviewing and editing the manuscript. PT was responsible for conceptualization, data collection, formal analysis, funding acquisition, investigation, methodology, project administration, resources, software, supervision, validation, and writing the original draft and reviewing and editing the manuscript. IIP was responsible for data collection, formal analysis, funding acquisition, investigation and methodology.

## Conflict of Interests

The authors declare no conflict(s) of interest related to the publication of this work.

## Acknowledgements

The authors declare no conflict of interest to this study

## Funding

This study was financially supported by the Research Fund of Mustafa Kemal University (Project number 18.M.028).

## Ethics approval

This study was approved by the Ethics Committee of Mustafa Kemal University.
